# Effects of an optimized dairy calf-rearing protocol on performance and health in the subsequent fattening period on Swiss veal farms

**DOI:** 10.1016/j.vas.2026.100590

**Published:** 2026-01-30

**Authors:** Julia Rell, Michael Walkenhorst, Mirjam Holinger, Corinne Bähler, Jens Becker, Martin Kaske

**Affiliations:** a Department of Livestock Sciences, Research Institute of Organic Agriculture (FiBL), Ackerstrasse 113, 5070 Frick, Switzerland; b Centre for Dam-Calf Contact Rearing, 8810 Horgen, Switzerland; c Swiss Calf Health Service, Vetsuisse Faculty, University of Zurich, Winterthurerstrasse 260, 8057 Zürich, Switzerland; d Clinic for Ruminants, Vetsuisse Faculty, University of Berne, Bremgartenstrasse 109a, 3012 Bern, Switzerland

**Keywords:** Veal calves, Intensive feeding, Preconditioning, Calf fattening, Antibiotics

## Abstract

We conducted a prospective field study to characterize the effects of an optimized rearing protocol of calves on dairy-farms on the subsequent performance, health and antimicrobial use (AMU) at the veal-farm. On 19 dairy-farms a total of 55 matched pairs of calves (same gender and breeding type, born within the same period and farm) were randomly assigned to the treatment group P (“preconditioned”) or C (“control”) during five two-week enrollment periods. The P-calves were fed milk ad libitum, received parenteral selenium and iron supplementation, wore calf jackets at low temperatures, and were vaccinated intranasally against bovine respiratory disease. The C-calves were fed restrictively and received no additional measures. At an average of 30 days of life, calves were transported directly to one of two veal-farms. Body weight and health score were determined between days 2 and 5 of life, at transport and in weeks 4 and 12 after arrival on the veal farm. All applications of antimicrobials were recorded. Mixed-effect models were applied for statistical analyses. While born with similar body weights, daily weight gain of the calves differed between groups on the dairy-farms (P: 1.10[0.96–1.25] vs. C: 0.70[0.55–0.84]kg/day; *p* < 0.001) but not on the veal-farms (P: 1.34[1.24–1.44] vs. C: 1.29[1.19–1.39]kg/day). A similar carcass weight was reached in shorter time for P-calves compared to C-calves (104[99–110] vs. 115[109–120] days on the veal-farm; *p* = 0.04). Neither clinical health nor AMU differed between the groups. Thus, while preconditioning was effective regarding performance parameters, a significant advantage in respect of health status and AMU could not be demonstrated.

## Highlights

• We tested the optimized rearing of calves on dairy farms.
• This optimized rearing improved daily weight gain on dairy farms.
• On veal farms these calves reached an optimal carcass weight in shorter time.
• However, neither clinical health was improved, nor antimicrobial use was reduced.

## Introduction

In Switzerland, around 190 000 veal calves are slaughtered per year (Proviande, 2024). Most of those calves are born on dairy farms and delivered to two types of fattening farms at an age of 3–6 weeks with a body weight (BW) of around 75 kg (Schweizer Kälbermästerverband, 2025). About two-thirds of these calves are fattened on small farms in the hilly and mountainous zones, where milk collection can be impossible. Then, the cow's milk is fed to calves for veal production (Lava et al., 2016a). Roughly 70,000 veal calves per year are fattened by companies in groups of 40–100 calves on farms on a fee basis using by-products of the dairy industry (predominantly whey; Bähler et al., 2016). Housing takes place in group pens with straw bedding («all in-all out system»). Milk feeding is accomplished by automatic calf feeders. Calves are fed for 12–20 weeks (Becker et al., 2020) a total of 250 kg dry matter from whole milk and/or milk replacer and/or milk by-products in order to achieve daily weight gains (DWG) of >1.35 kg throughout the fattening period. Calves are slaughtered at a BW of 220–240 kg to harvest a carcass of 120 kg (Proviande, 2024).

Transport and regrouping of calves at an early age and within the so-called immunological gap approximately between two and eight weeks of life (Chase et al., 2008) is accompanied by an increased risk of disease in the subsequent fattening period on a veal farm. In particular, morbidity and mortality due to BRD and digestive disorders are high in the first weeks on the veal farm (Pardon et al., 2013; Lava et al., 2016a; Schnyder et al., 2019). This causes significant use of antimicrobials (AMU) which enhances the development of antimicrobial resistance and therefore represents a significant global health challenge (Prestinaci et al., 2016). In Switzerland, a national monitoring program quantifies the amount of antimicrobial compounds used in farm animals. The current annual report reveals that the proportion used for fattening calves accounts for at least 38 % of the total amount used for cattle (Jahresbericht, 2024). Antimicrobials are administered to calves primarily as metaphylactic oral group medications (Lava et al., 2016a), which is defined as simultaneous treatment of clinically healthy and diseased animals in the same pen or group (Schnyder et al., 2019).

Strategies to improve calf health and reduce AMU on different levels have been proposed in the past years. Fattening only the calves from the own dairy herd, i.e. completely abandoning transport and regrouping, is a powerful leverage factor as shown by Lava et al. (2016a). However, dairy farmers' willingness to switch to this system is limited (given reasons: no space; no time; preference to stay specialized in milk production) and strongly depends on financial incentives (Becker & van Aken, 2021). Effects of a novel concept (based on direct transport to partner veal farms, vaccination against pneumonia upon arrival, a three-week quarantine in individual hutches and outdoor-fattening in small groups) are promising regarding the reduction of AMU (Becker et al., 2020). A widespread changeover to this system would require cost coverage of the proposed housing system. System changes are subject to considerable structural challenges (Rell et al., 2020), which cause a prolongation of the process. Accordingly, approaches are needed which do not require a system change.

One option is improving the robustness and functional efficiency of the immune system by optimizing the rearing protocol of the calves on the dairy farm prior to transport. Upstream measures to improve the health status prior to transport to the fattening farm are known as «preconditioning». Adequate feed intake and nutrition are essential for the immune function in cattle because immune cells require substantial energy and nutrient substrates for proliferation, antibody production, and pathogen defense (Hersom, 2014) which makes BW in combination with other factors such as age or breed a reliable indicator for robustness (Marcato et al., 2022). Further, a high feeding intensity during the first weeks of life allows the full growth potential to be realized (Hilton, 2015; Frieten et al., 2017) and affects the long-term metabolic performance of the adult organism (Maccari et al., 2015; Prokop et al., 2015; Schäff et al., 2018). This phenomenon, called ‘nutritional programming’, ‘developmental programming’, or ‘metabolic imprinting’ (Guilloteau et al., 2009; Kaske et al., 2010), permanently affects the release of hypothalamic neuropeptides controlling feed intake and long-term weight gain due to the plasticity of the regulatory system (Taylor & Poston, 2007). Accordingly, an intensive feeding regime at the dairy farm might not only enable a strong body condition before transfer to the veal farm (Renaud & Pardon, 2022) but also affect the long-term weight gain and age at slaughter. However, studies focusing on preconditioning have been performed mostly in the US, where the productions system differs markedly from those used in Europe.

Next to nutritional programming, supplementation of iron, selenium and vitamin E can improve calves' immune system by enhancing the oxidative metabolism with protective effects on diarrhea incidence (Salles et al., 2025). Regardless of vaccination timing and vaccine type, one Swiss study reported a significant protective effect of vaccination against calf pneumonia indicating higher mortality in unvaccinated calves (Lava et al., 2016b). In contrast, another study found the opposite association, showing significantly higher treatment intensity in herds where calves were vaccinated against pneumonia. This was attributed to reverse causality, as vaccination was more likely implemented in herds with a higher underlying burden of respiratory disease and where herd managers had already introduced control measures, including calf vaccination (Schnyder et al., 2019). Intranasal vaccination with life attenuated bovine respiratory syncytial virus and bovine parainfluenza 3 virus is a common measure on Swiss veal farms (Lava et al., 2016b). Calf jackets act as a protective barrier against adverse environmental conditions (Rutherford et al., 2019). Neonate calves have little body fat reserves causing poor abilities to cope with cold-stress (reviewed by Roland et al., 2016). Though scientific results regarding the effect of calf jackets on calf health are contradictive (Scoley et al., 2019; Rutherford et al., 2020; Çam et al., 2024), many Swiss dairy farmers routinely use them during the winter months (Rell et al., 2020; data not shown) and are generally open adopt measures that are easy to implement (Rell et al., 2020). This has also been shown in the survey-based study by Becker and van Aken (2021), where “taking specific health measures on the source dairy” received the highest proportion of agreement (40–55 %; depending on the proposed bonus of 10 to 75 CHF per calf) compared to other proposed strategies. Indeed, various members of the industry introduced such programs supported by the Swiss Calf Health Service (Schweizer Bauernverband, 2018). Therefore, scientific studies are needed to evaluate the actual value of this preconditioning approach. In this study we aimed to test the effects of an “optimized rearing protocol” consisting of ad libitum feeding of milk or milk replacer, vaccination against BRD, parenteral substitution of iron, selenium and vitamin E and the use of calf jackets at low temperatures. The research questions to be answered are: Does the application of the optimized calf rearing protocol 1) improve calves' health status determined by mortality, clinical health, red and white blood cell counts and AMU in the subsequent fattening period and 2) enhance fattening performance determined by DWG, duration of the fattening period and carcass quality.

The hypothesis to be tested was that the application of the optimized calf rearing protocol improves the calf health status and fattening performance and reduces AMU during the subsequent fattening period compared with the standard rearing practice (exact protocols are described in the material and methods section).

## Material and methods

### Study design

A prospective randomized controlled field study was conducted in Switzerland (CH) on 19 commercial dairy farms and 2 veal farms. The study was approved according to Swiss legal requirements by the cantonal veterinary office Aargau (Ag 75,706).

After a public announcement of the project, all farmers interested to participate in the study were contacted and visited. Selection criteria for the dairy farms were (a) >80 dairy cows, and (b) being located within a distance of <2 h by car to the research institute in Frick, Switzerland. Ultimately, 19 farms were selected. Thirteen farmers kept herds with more than one breed, mostly Holstein Friesian (HF), Red Holstein (RH), and Brown Swiss (BS) cows. Five herds had exclusively HF cows and one farm only had BS cows. The average herd size was 120 cows (minimum 80, maximum 210 cows).

**Fig. 1:**
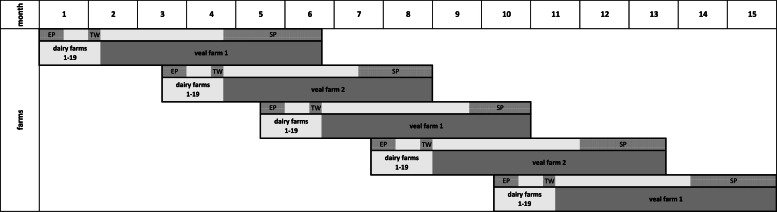
Time schedule of the five trial runs (starting in December 2017) with duration on the dairy farm (light gray) and on the veal farm (dark gray). EP indicates the two-week enrollment phase of each run. TW refers to the week of transport (five days) in which calves left the dairy farms and were moved to the veal farm. SP indicates the period of gradual slaughter of the calves.

On the dairy farms, calves were selected which were born within five two-week enrollment periods (five runs) between December 2017 to October 2018 (Fig. 1). Within these two-week intervals, matched pairs of calves were made using a randomized block design: each calf pair consisted of calves of the same gender, same breed type (either pure milk type [M, i.e. dam and sire HF, RH or BS] or beef-crossbred type [B; i.e. HF, RH or BS dam and beef sire]) and born immediately after each other on the same farm. Within a pair each calf was assigned randomly either to the treatment group (preconditioning; P) or the control group (control; C).

### Procedures on dairy farms

Farmers were instructed to offer colostrum to all calves ad libitum as soon as possible, at least twice within the first 12 h of life. Independent of being in the P- or in the C-group, all calves were housed outdoor during their first three to five weeks of life on the dairy farms in single calf hutches with straw bedding. Hay and water were available ad libitum. Preconditioning for the P-calves comprised (a) ad libitum feeding of colostrum at the first day of life and milk or milk replacer from the second day of life and onwards, (b) parenteral application of vitamin E and selenium supplementation (Tocoselenit®; 0.2 mg sodium selenit and 5 mg α-tocopherol/kg BW; Gräub AG, Berne, Switzerland) and iron (Belfer®, 1 g iron as iron-III-dextrane; ufamed AG, Sursee, Switzerland) between day 2 and 5 of life, (c) vaccination with an attenuated intranasal vaccine against BRSV and Parainfluenza-3 (Rispoval® RS + PI3 Intranasal, Zoetis, Delémont, Switzerland) between day 8 and 12 of life, (d) calf jackets (Albert Kerbl, Buchbach, Germany) for the first two weeks of life at environmental temperatures below 15 °C.

The calves of the C-group were fed colostrum ad libitum at the first day of life and milk or milk replacer from the second day and onwards restrictively (2.5 L twice per day in the first week of life; 3 L twice per day in the following weeks) and received neither a vitamin E‐, selenium‐ or iron supplementation nor a vaccination or calf jacket.

The veterinarians serving the dairy farms were informed about the research activities and the recommended treatment protocol for calf diseases (Supplement 1). On all dairy farms, a test run was performed in October 2017 to train the dairy farmers with respect to reporting calving, documentation, and consideration of the two different rearing protocols (P or C).

### Procedures on veal farms

Two veal farms (#1 and #2) producing according to label standards for high animal welfare (“Naturafarm”; Coop, 2004) and practicing the all-in-all-out stocking system were chosen for the study. The calves were group-housed on clean and dry non-perforated floors with deep straw bedding at a stocking density of > 3.5 m^2^ per calf, free access to an uncovered, non-bedded outside pen with concrete flooring, and availability of hay and water ad libitum.

Veal farm #1 had four pens for up to 50 calves each and veal farm #2 had two pens for up to 50 calves each. For this study, one of the pens was halved to enable the housing of up to 25 calves per pen. Each of the pens was divided from other compartments by a solid wall to prevent nasal contact while maintaining shared air space. The calves in each pen had access to an automatic milk feeder where commercial milk replacer was offered ad libitum.

Veal farm #1 performed three and veal farm #2 two runs within this study (Fig. 1). The pens on each farm were switched between the P- and C- groups for each new fattening period to minimize environmental effects from the pen.

Calves of each run were brought together from the different dairy farms to the veal farm within 2 days. Transport occurred directly. The calves of group P and C were transported separately in different trailers. On the day of arrival at the fattening farm, the C-calves were vaccinated with an intranasal vaccine against Parainfluenza-3 virus and Bovine Respiratory Syncytial Virus (Rispoval® RS + PI3 Intranasal, Zoetis, Delémont, Switzerland). About one week later, the P-calves were boostered with the same vaccine. Prior to the application of the vaccine, rectal temperature of each calf was measured. Calves exceeding a body temperature of 39.4 °C were skipped and vaccinated three days later after normalization of temperature.

Regular meetings were performed with the responsible veterinarians of the two veal farms during the first weeks after stabling new calves to ensure compliance with treatment recommendations (Supplement 1) and to discuss necessary deviations. As the trial was performed under field conditions, an oral group treatment with antimicrobials was administered in all groups within the first two weeks after arrival. This was the standard practice on the two veal farms. The treatment consisted of 30 g of a amoxicillin premix (Amoxan 70®, ufamed AG, Sursee, Switzerland) per 100 kg BW which was administered using a medication dispenser of the automatic feeder for a period of 7 to 10 days (i.e., 21 mg amoxicillin per kg BW per day).

### Data collection

Data were collected during regular farm visits from all calves at an individual level. A commercial data management software (Microsoft Access®, Microsoft, Redmont, WA, USA) was used to organize the various data sets of the calves and farms. The on-farm observation ended in week 12 of fattening as from this timepoint on a notable number of calves were slaughtered. The slaughter date of each individual animal was decided by the farmer based on an estimated slaughter weight, regardless of the age of the calves. The maximal target slaughter weight for veal calves in Switzerland is 130 kg, as there is a significant pay deduction for higher carcass weights. Carcasses were classified according to the Swiss assessment system for conformation and fat coverage (CH-TAX; Proviande, 2025).

The clinical status of each calf was evaluated at an age of 2 to 5 days, 8 to 12 days, at the day of transport from the dairy farm to the fattening farm, and in weeks 1, 2–3, 4, and 12 on the fattening farm by two veterinary researchers according to a scoring system. A detailed description of the used scoring system was formerly published in the study of Ayrle et al. (2021). The protocol included the general health status (posture, vitality, nutritional state), the rectal temperature (VT 1831, Microlife AG Swiss Corporation, Widnau, Switzerland), the respiratory tract (respiratory rate, respiratory intensity, auscultation, nasal discharge, coughing), the gastrointestinal tract (consistency, color, and abnormal admixtures in the feces), the position of the ears, bulb position (enophthalmus), episcleral vessels, and the navel and joints (increased circumference or temperature, signs of pain).

Blood samples were collected, and the BW was determined at an age of 2–5 days, on the day of transport from the dairy farm to the veal calf farm, and in weeks 4 and 12 on the veal farm. Blood was drawn from the jugular vein after cleaning the puncture site (alcohol 70 % w/v). The blood samples (3 ml) were stabilized using K3-EDTA tubes (Sarstedt AG, Nürnberg, Germany). The blood tubes were cooled (4 °C) after collection until a hematogram, including white blood cell counts and hemoglobin concentration (Hb), was made within 24 h after sampling using a flow cytometer (XT-2000, Sysmex Digitana AG, Horgen, Switzerland). A second blood sample (9 ml) was drawn at an age of 2–5 days to assess the serum concentration of total protein after centrifugation (Kestrel Compact, MSE, United Kingdom). Total serum protein was determined optically with a brix refractometer (Model RF10, Extech, Nashua, NH, USA). The BW was assessed with a portable digital scale (Nohlex GmbH, Buchholz, Germany) (Table 1).

**Table 1:** Abbreviations as used in this article.

Term	Abbreviation
Antimicrobial use	AMU
Beef-crossbred type	B
Body weight	BW
Bovine respiratory disease	BRD
Brown Swiss	BS
Control	C
Daily weight gain	DWG
Hemoglobin concentration	Hb
Holstein Friesian	HF
Pure milk type	M
Preconditioned	P
Red Holstein	RH

On the dairy farm, milk or milk replacer intake, health status, mortality and medical treatments were recorded daily by the farmers using a standardized protocol. To ensure objective decisions, all farmers used the same scoring sheets based on a standardized assessment key (supplement 1). Rectal temperature was measured whenever any signs of illness were observed.

On the veal farm, the general health status of each calf was assessed daily by the farmers. Farmers were not aware which group had received the improved rearing protocol. Any deviations as well as fatalities were recorded on a scoring sheet based on a standardized assessment key (supplement 1). The observers were, however, not blinded to treatment, as they transported calves from the dairy to the veal farms. Objective decisions on medical treatments were ensured by introducing an assessment key with defined conditions for starting a treatment. Drugs and doses for individual treatments were preset by a therapy plan (supplement 2) depending on the symptoms (signs of infection of the respiratory tract, gastrointestinal tract, ears, navel, or joints) according to the recommendations of the Swiss Calf Health Service.

After slaughter, reports of performance data were obtained from the abattoirs. For each calf, the data sheet included slaughter date, carcass weight, conformation class according to the Swiss CHTAX system, fat coverage (based on a scoring system between 1 [without any fat cover] and 5 [massively obese]) and the l-value (lightness; arbitrary units) indicating meat color (determined by a chromameter [Minolta Chroma-Meter CR 410, Konica-Minolta Europe, Langenhagen, Germany]). Payment deductions incurred (a) if calves were slaughtered at an age of >160 days, and (b) if the l-value was < 39 (age at slaughter < 160 days) or < 42 (age at slaughter > 160 days), respectively.

### Calculation of AMU

For each calf, the number of days under effective levels of antimicrobials due to either oral group medication or parenteral individual treatment was calculated by multiplying the number of administrations during an individual treatment with the effective duration of the drug used. For the effective duration, data from the Swiss Compendium of Veterinary Pharmaceuticals (CliniPharm, Vetsuisse Faculty, University of Zurich, 2021) were used.

### Statistics

Statistical analyses were performed using the software R (Version 4.1.0; R Core Team, 2021). Initial birth weight was compared between treatment groups with a *t*-test. (Generalized) linear mixed effect models with the functions “glmer” and “lmer” from the package “lme4” were applied (Bates et al., 2015) to model the outcome variables. Outcome variables, fixed effects, random effects, as well as transformations of outcome variables are presented in Table 2. Carcass characteristics (high carcass quality: C, H, and *T*+ = 1; low carcass quality: T, T-, A, *X* = 0), fat coverage (optimal fat coverage: score 3 = 1; undesirable fat coverage: scores 1, 2, 4 and 5 = 0) and antimicrobial treatment (yes/no) were analyzed as binary variables. The random effect structure differed between outcome variables related to the dairy farms and outcome variables that had been measured during the fattening period. On the dairy farms, calves were housed individually. On the fattening farms, calves were housed in groups. These dependencies among calves of the same farm or same group were corrected by using crossed random effects. The nested random effects reflected the experimental design with repeated measurements per animal, pair, and run. Total serum protein was included as a covariable in all models concerning data collected on the dairy farms. It was omitted from models assessing outcomes on the veal farm, reflecting the unknown biological relevance of this early parameter in predicting performance and health over the extensive fattening period. Model assumptions were tested by visually inspecting model residuals for deviations from normality or homogeneity of variance. If deviations were detected, outcome variables were transformed with either a log, log link (poisson distributed data), or a logit link (binomial data) function as displayed in Table 2. Due to the overdispersion of the health score data, the single observation was included as a crossed random effect.

**Table 2:** Description of the applied (generalized) linear mixed models; TP = Total protein, Hb = hemoglobin concentration.

Outcome variable	Fixed effects^1^	Random Effects	Transformation
Health score on dairy farm	Treatment * Breed type + Treatment * SamplingNr + TP	Nested: Measurement in animal in pair in run Crossed: Dairy farm, single observation	Log link function
Health score on veal farm	Treatment * Breed type + Treatment * SamplingNr	Nested: Measurement in animal in pair in run Crossed: Group, single observation	Log link function
Hb on dairy farm	Treatment * Breed type + Treatment * SamplingNr + TP	Nested: Measurement in animal in pair in run Crossed: Dairy farm	None
Hb on veal farm	Treatment * Breed type + Treatment * SamplingNr	Nested: Measurement in animal in pair in run Crossed: Group	None
Antimicrobial treatment on dairy farm	Treatment * Breed type + TP	Nested: Measurement in animal in pair in run Crossed: Dairy farm	Logit link function (binary data)
Antimicrobial treatment on veal farm	Treatment * Breed type	Nested: Animal in pair in run Crossed: Group	Logit link function (binary data)
Body weight at transport to veal farm	Treatment * Breed type + Treatment * Body weight at birth + TP	Nested: Measurement in animal in pair in run Crossed: Dairy farm	None
Duration of fattening period	Treatment * Breed type	Nested: Animal in pair in run Crossed: Group	None
Daily weight gain on dairy farm	Treatment * Breed type + Treatment * Body weight + TP	Nested: Measurement in animal in pair in run Crossed: Dairy farm	None
Daily weight gain on veal farm	Treatment * Breed type + Treatment * Period	Nested: Period in animal in pair in run Crossed: Group	None
Carcass weight	Treatment * Breed type	Nested: Animal in pair in run Crossed: Group	None
Carcass classification	Treatment * Breed type	Nested: Animal in pair in run Crossed: Group	Logit link function (binary data)
Fat coverage	Treatment * Breed type	Nested: Animal in pair in run Crossed: Group	Logit link function (binary data)

1 A star (*) between fixed effects means that, next to the main effects, also their interaction was assessed.

We encoded all factorial fixed effects as dummy variables with sum contrasts. P-values were calculated by comparing the full model to a reduced model without the respective main effect or the interaction. The comparison was performed by using a parametric bootstrap approach (function “PBmodcomp”; package “pbkrtest”; Halekoh & Hojsgaard, 2014). If the comparison full model versus the null model (without any fixed effects) resulted in a p-value above 0.1, no further calculations were carried out. Model estimates and confidence intervals were obtained similarly with parametric bootstrap simulations (function “bootMer”; package “lme4”; Bates et al., 2015). We reported model estimates and confidence intervals for all variables that were statistically tested and means and standard deviations for descriptive results.

The total protein measurement was missing for one calf. Therefore, this calf and its paired partner calf were omitted for analyses concerning the effects on the dairy farm. Two outlier data points had to be left out due to their very strong influence on the model: Body weight at transport of 125.7 kg and Hb of 3.9 mmol/l. No model was calculated for the variable l-value (binary variable according to payment deduction schedule: either < 39, if age at slaughter is < 160 days, or < 42, if age at slaughter is >160 days) and mortality because of very low prevalences.

## Results

A total of 178 calves from 19 dairy farms were enrolled in the trial and 55 matched pairs from 14 dairy farms were ultimately used (i.e., 110 calves; Table 3). Sixty-eight additional calves could not be matched with another calf and were excluded from the analyses. Ninety-five of the remaining matched-pair calves (86.4 %) were born from pluriparous cows and 15 calves (13.6 %) were born from primiparous cows.

**Table 3:** Characteristics of the 110 calves from the 55 matched pairs; TP - total serum protein at the date of enrollment.

	Preconditioned (P) (*N* = 55)	Control (C) (*N* = 55)
Male beef-type	22	22
Male milk-type	15	15
Female beef-type	13	13
Twins	5	5
Calves from primiparous cows	8	7
Calves from multiparous cows	47	48
TP < 55 g/L	19	21

### Performance

Body weight on the first day of life was similar in both groups (mean ± SD; P: 46.5 ± 7.9 kg; C: 45.4 ± 6.8 kg; *p* = 0.43) and type (M: 46.2 ± 5.7; B: 45.9 ± 7.9 kg). The mean total milk intake on the dairy farms was 303 + 100 L for P-calves and 177 + 60 L for C- calves. The age of the calves at transport from the dairy farm to the veal farm was similar for calves in the P- and C-group (29.8 ± 5.5 *vs.* 30.5 days ± 5.2 days).

On the dairy farms, the DWG of the calves was higher for the P calves (model estimate [95 % confidence interval]; 1.10 [0.96–1.25] kg/d; Table 4) than for the C calves (0.70 [0.55–0.84] kg/d; *p* < 0.001). DWG was found to be higher for calves with a higher BW at birth (*p* = 0.02) and with a higher serum TP concentration (*p* < 0.01).

**Table 4:** Model estimates and 95 % confidence intervals for the daily weight gain (kg/d) of the preconditioned calves (P) and control calves (C).

Treatment	Breed type	N	Dairy farm^1^	Veal farm^2^		
				Week 1 – 4	Week 5 – 12	Week 1 – 12
P	All	55	1.10 [0.96–1.25]	1.17 [1.07–1.27]	1.51 [1.41–1.62]	1.34 [1.24–1.44]
	Milk-type	14	1.09 [0.90–1.26]	1.15 [1.00–1.29]	1.49 [1.34–1.63]	1.32 [1.17–1.47]
	Beef-type	41	1.12 [0.97–1.25]	1.19 [1.08–1.29]	1.53 [1.42–1.64]	1.36 [1.26–1.46]
C	All	55	0.70 [0.55–0.84]	1.11 [1.00–1.22]	1.48 [1.38–1.59]	1.29 [1.19–1.39]
	Milk-type	15	0.68 [0.49–0.86]	1.08 [0.94–1.23]	1.45 [1.31–1.60]	1.27 [1.13–1.41]
	Beef-type	40	0.71 [0.56–0.86]	1.13 [1.02–1.24]	1.50 [1.40–1.61]	1.32 [1.22–1.42]

1 Data from the dairy farms and veal farms were analyzed in separate models due to differences in random effect structure. Model results: Treatment (*p* < 0.001), body weight at birth (*p* = 0.02), total serum protein (*p* < 0.01), breed type (*p* = 0.62), treatment * breed type (*p* = 0.95), treatment * body weight at birth (*p* = 0.35).

2 Data from the dairy farms and veal farms were analysed in separate models due to differences in random effect structure. Model results: Treatment (*p* = 0.37), breed type (*p* = 0.48), period (*p* < 0.001), treatment * breed type (*p* = 0.92), treatment * period (0.60).

On the day of transport to the fattening farm and in weeks 4 and 12 of the fattening period, the BW of the P-calves was 12–14 kg higher than that of the C-calves (mean ± SD; day of transport: 78.7 ± 15.3 *vs*. 66.7 ± 12.0 kg; fattening week 4: 112.4 ± 21.2 *vs.* 99.3 ± 16.8 kg; fattening week 12: 189.3 ± 31.9 *vs.* 175.1 ± 25.2 kg).

On the veal farm, neither the treatment group (P vs. C; *p* = 0.37) nor type of the calf (M vs. B; *p* = 0.48; Table 4) affected DWG and there was no interaction between treatment and breed type (*p* = 0.92). The DWG was significantly lower in the first 4 weeks of the fattening period than between weeks 5 and 12 (*p* < 0.001). From the day of transport until week 12 of fattening the DWG of the P-calves averaged 1.34 kg (CI: 1.24 – 1.44) and 1.29 kg (CI: 1.19 – 1.39) for the C-calves.

Duration of the fattening period was 10.4 days shorter for P-calves (104.3 days) than for C-calves (114.7 days; *p* = 0.04; Table 5). Breed type alone had no effect on the fattening duration, but a statistical interaction was found between treatment and breed type (*p* = 0.08) i.e., the effect of preconditioning was slightly more pronounced in the M- than in the B-calves (Table 5).

**Table 5:** Model estimates and 95 % confidence intervals for duration of the fattening period of the preconditioned calves (P) and the control calves (C).

Treatment	Breed type	N	Fattening period^1^ [days]
P	All	55	104.3 [98.8–110.1]
	Milk-type	14	104.7 [97.2–112.3]
	Beef-type	41	103.9 [98.5–109.1]
C	All	55	114.7 [109.3–120.2]
	Milk- type	15	119.5 [111.6–126.7]
	Beef-type	40	109.9 [104.7–115.7]

1 Model results: Treatment (*p* = 0.04), breed type (*p* = 0.14), interaction between treatment and breed type (*p* = 0.08).

### Carcass quality

The mean age at slaughter was 134 + 14 days for the P-group calves and 143 + 14 days for the C-group calves. Six C-group calves (5 M and 1 B) and one P-group calf (B) exceeded the age of 160 days at slaughter which caused pay deductions. The estimated carcass weight of the veal calves was 124.9 kg [CI: 120.1–129.7] for the C calves and 125.1 [CI: 120.5–129.9] for the P calves and was neither affected by the breed type (M vs. B) nor the treatment (P vs. C; comparison of full to zero model *p* = 0.27).

The preconditioning treatment had no effect on carcass classification (*p* = 0.36). However, the breed type had an effect. While 91 % of the B-calves were classified in the superior classes C, H, and *T*+, the respective proportion of the M-calves was only 14 % (*p* < 0.001).

Fat coverage of the carcass was neither affected by breed type (M vs. B) nor by treatment (P vs. C; comparison of full to zero model *p* = 0.22). Payment deductions due to meat color applied to two calves of the C-group, whereas no calf of the P-group was affected. The mean of the l-values for group P was 44.8 ± 2.6 and 45.7 ± 2.9 for group C.

### Health scores and blood parameters

During the five enrollment periods and the subsequent weeks until transport to the veal farms, no calf died. On the veal farms, three C-group calves (1 M-calf, 2 B-calves) died or were euthanized representing a mortality rate of 5.5 % while no fatalities were observed in the P-group.

None of the tested variables affected the health scores of the calves on the dairy farms (comparison of full to zero model *p* = 0.26). During the fattening period, breed type had an effect on the health scores of calves as the B-calves revealed higher scorings (lower health status) than the M-calves (*p* < 0.01).

The serum total protein concentration at enrollment was similar between the groups (P: 56.9 ± 6.6; C: 57.3 ± 8.5 g/L) with values below 50 g/L in six P-calves and nine C-calves, respectively.

The Hb of the calves on dairy farms was affected by treatment (*p* < 0.001), age (*p* < 0.001), serum total protein (*p* = 0.01), an interaction between treatment and day of life (*p* < 0.001), and breed type (*p* = 0.03). The detected association between total protein concentrations at enrollment and Hb concentration was positive. The M-calves showed lower Hb values than the B-calves. While in the P-calves the Hb concentration remained on a similar level from birth to transport, it decreased in the C-calves (Table 6).

**Table 6:** Model estimates and 95 % confidence intervals for hemoglobin concentration (Hb) [mmol/L] of the preconditioned calves (P) and the control calves (C).

Treatment	Sampling number	Hb [mmol/L]
P	Enrollment^1^	6.68 [6.26–7.07]
	Day of transport^1^	6.75 [6.32–7.14]
	Week 4 on veal farm^2^	6.28 [5.94–6.62]
	Week 12 on veal farm 2	6.36 [6.02–6.71]
C	Enrollment^1^	6.33 [5.94–6.73]
	Day of transport^1^	5.51 [5.12–5.92]
	Week 4 on veal farm 2	5.64 [5.32–5.94]
	Week 12 on veal farm 2	6.41 [6.09–6.73]

Data from dairy farms and veal farms were analysed in separate models due to differences in random effect structure.

1 Model results for dairy farms: Treatment (*p* < 0.001), breed type (*p* = 0.03), sampling number (*p* < 0.001), total serum protein (*p* = 0.01), interaction treatment * breed type (*p* = 0.76), interaction treatment * sampling number (*p* < 0.001).

2 Model results for veal farms: Treatment (*p* < 0.001), breed type (*p* < 0.001), sampling number (*p* < 0.001), interaction treatment * breed type (*p* = 0.18), interaction treatment * sampling number (*p* < 0.001).

On the veal farms, Hb was affected by the preconditioning treatment (*p* < 0.001), fattening period (*p* < 0.001), an interaction between treatment and fattening period (*p* < 0.001), and breed type (*p* < 0.001). As on the dairy farm, the B-group calves had higher Hb concentrations than the M-group calves. At 12 weeks on the veal farm, P- and C-calves had similar Hb concentrations.

None of the tested variables had an effect on the number of white blood cells of the calves on the dairy farms. On the veal farms, the white blood cell count was higher in week 4 than in week 12 (*p* < 0.01).

### Antimicrobial treatments

For the antimicrobial oral group treatments either amoxicillin or doxycyclin was used. The numbers of days under oral group treatment is shown in Table 7. The main indication for individual treatments was BRD. Sixtysix percent of the individual treatments were performed with oxytetracyclin and florfenicol accounting for 144 and 292 days under individual treatment, respectively. Other active substances used were gentamicine, danofloxacin or a combination of dihydrostreptomycine and procaine penicillin. On the dairy farms, about 10 % of the calves were treated with antimicrobials. On the veal farms, calves got on average 12 days of oral antimicrobial treatments. In addition, more than half of the calves were treated individually during the fattening period.

**Table 7:** Use of antimicrobials during rearing of the preconditioned calves (P) and the control calves (C) on the dairy farm and during the fattening period on the veal farm.

Variable	Level	Group P	Group C	p-value
Proportion of treated calves	Dairy farm	0.11 [0.00–0.22]^1^	0.10 [0.00–0.20]	0.88
Days under group treatment	Veal farm	11.3 ± 5.0	12.4 ± 6.4	-
Days under individual treatment	Veal farm	4.5 ± 5.3	6.0 ± 6.9	-
Total days under treatment (group and individual)	Veal farm	15.8 ± 5.4	18.4 ± 7.9	-
Proportion of individually treated calves	Veal farm	0.63 [0.32–0.97]	0.63 [0.29–0.90]	0.96

1 Model estimates and 95 % confidence intervals are provided for the variables that were analyzed statistically (p-value shown). Mean ± SD are provided for all other variables.

## Discussion

### Experimental design

Although complex, the design of the study allowed a reliable assessment of the impact of the applied rearing protocol on the subsequent performance and AMU in veal calves. Performing the study with matched pairs resulted in a balance regarding gender and M and B calves in the P- and C-groups. One important benefit of the matched pair-approach is that within pairs, dairy farm- and time-related confounders such as the hutch-cleaning routine or the temperatures during the first weeks were eliminated. Accordingly, any interference with the results of these factors affecting performance and health status was avoided. Regarding our approach to blind the veal farmers to treatment, the difference in BW at arrival disabled the blinding. However, as treatments the data we collected were not based on the farmers assessments, but on our measurements and the treatment protocol, negative effects of deblinding can be excluded. The main shortcoming of the study is that only the effects of the rearing protocol as a whole could be evaluated. A much more complex study design (individual treatment and control groups for each of the applied measures) would have been necessary to assess this. Our objective in this study was to test a set of feasible on-farm measures that is realistic in the landscape of Swiss dairy and veal farming (see Introduction), and not to provide evidence of each measure's effectiveness. The oral group metaphylaxis on arrival at the veal farms can as well be seen as a study limitation as differences between the study groups might have been more pronounced without this group treatment. However, oral metaphylactic group medications over 6–10 days using preferably doxycycline or amoxicillin are widely applied (Baptiste & Kyvsgaard, 2017) and administered on almost all professional fattening farms in Switzerland (Gallin-Anliker et al., 2021). We had no option to circumvent this treatment as both farmers were not willing to deviate from their standard practice. Given reasons were expectation of increased health problems or calf losses which they would have had to compensate at their own expense.

### Rearing protocol

The highly significant differences in DWG on the dairy farm (Table 4) indicate that the farmers implemented the different treatment protocols and followed the study protocol. Interestingly, even in the C-calves, a moderate DWG was achieved, being superior to other commercial dairy farms in Switzerland. This implies overall good conditions on the dairy farms included in this study. Also, the administration of iron resulted in significant differences in the Hb between the groups at transport to the fattening farms which may have contributed to the higher DWG observed in the P-calves compared to the C-calves (Mohri et al., 2010; Hecker et al., 2022). However, it was not possible to achieve appropriate colostrum supply for all the calves although we had emphasized the major importance of this measure to the farmers. In fact, in the group of P-calves as well as in the C-calves, roughly 40 % had serum TP concentrations of <55 g/L indicative of a partial failure of passive transfer (McGuirk & Collins, 2004; Godden, 2019). This finding was in agreement with results from comparable Swiss dairy farms (Reschke et al., 2017). Having in mind that failure of passive transfer or low levels of immunoglobulin G are associated with increased morbidity and mortality rates and lower DWG of calves (reviewed by Cuttance et al., 2018), it remains open whether this major constraint represents an explanation for the unexpectedly high medication rates of even the P-calves on the veal farm.

### Production data

The intensively fed P-calves achieved an advance of 10.5 kg BW during their first 30 days of life compared to the C-calves while consuming 123 L more milk. Consume as well as DWG of P-calves were within the expected frame and in agreement with Maccari et al. (2015), where ad libitum-fed calves consumed 10.8 kg milk/day in the second and third week of life gaining 1.3 kg/day (mean over the first three weeks of life). However, in our study even the M-calves of the P-group achieved a DWG well above 1 kg which is higher than usually in pure dairy breeds (Maccari et al., 2015; Prado et al., 2021). This is probably due to even more restrictive feeding protocols in the other studies. The additional expenditure of the dairy farmer, in particular 123 kg more milk or milk replacer feeding, must also be taken into account and remunerated.

The first weeks on a veal farm represent an especially challenging period for calves as they have not only to cope with the stress of transport and regrouping, but they also have to adapt to an unknown feeding system and a new feeding regime. Growth depression, as seen in both of our study groups is common during this period, the health status had been stabilized and the calves were able to realize their maximal growth potential. Interestingly, based on the entire life span, the DWG of the P-calves was 16 % higher compared to the C-calves (1.26 *vs.* 1.09 kg) – clearly indicating the advantage of intensified milk-feeding during the first weeks of life. Furthermore, (a) the P calves needed 10 days less than the C calves to achieve the desired carcass weight, (b) deductions for calves due to late slaughter or reddish meat arose for 8 C-calves but only 1 P-calf and (c) three C-, but no P-calves died or where euthanized on veal farms. In the overall view, this assumption is in accordance with recently published Dutch studies, where the robustness of calves depended predominantly on the BW of the calf at arrival on the veal farm (Renaud et al., 2018; Marcato et al., 2022).

The breed type, but not the treatment significantly affected the classification of the carcass according to the Swiss quality assessment system (CHTAX) indicating that genetic differences between M and B calves were not affected by the different feeding regimes during the first weeks of life.

### Antimicrobial use

The average number of days under group medications on the veal farm was 11.3 and 12.4 in the P- and C-calves, respectively. In other studies, days under group treatment per fattening cycle were 30 (Beer et al., 2015) and 12 (Räber et al., 2013). The number of group medications per run was in line with results of other studies (Beer et al., 2015; Gallin-Anliker et al., 2021). International studies cite similar numbers for AMU in veal production in Canada (Scott et al., 2019), and Belgium (Bokma et al., 2019; Jourquin et al., 2022).

No statistically significant differences regarding health status, days under group treatment or proportion of individually treated calves were found between P- and C-calves on the veal farms. This result is according to another study that demonstrated the reduction in non-antimicrobial, but not in antimicrobial treatments for calves at a higher BW at arrival on a veal farm (Marcato et al., 2022). Preconditioning calves to achieve sufficient immunological robustness to withstand the load of pathogens and stress coming with the shift to the fattening period seemed to be not successful by implementing the optimized rearing protocol. We addressed the main risk factors for increased morbidity, mortality or AMU in dairy calves (e.g. Gorden & Plummer, 2010; Mee et al., 2023). However, given the importance of passive transfer for future health, the low TP values in both groups must be considered at this point. Possibly, enforcing prompt and sufficient colostrum intake (which was not possible due to the experimental setting in this study) would have affected the results. The stress load due to transport in our study can be assumed to be less than usual (transport duration < 2 h and commingling only with calves in the same fattening group). Thus, optimizing the calf rearing conditions on the dairy farms may not be powerful enough to enable a body constitution that can withstand the stressors coming with the veal fattening system. Risk factors for morbidity and consequently AMU or mortality in Swiss veal farming include regrouping, large group size, shared air space of various groups, poor barn climate and stocking density (Lava et al., 2016a, 2016b; Schnyder et al., 2019). Elimination of these factors within the existing branch of large-scope veal fattening requires substantial structural changes as shown by Becker et al. (2020). Small-scale veal farming, as still widely practiced in Switzerland, or even a shift towards veal fattening on the dairy farms of origin (Lava et al., 2016a)have more potential to reduce AMU than our current approach. For example, Schnyder et al. (2019) found a median overall treatment incidence of 2.1 days per animal year in small farms and 26 days per animal year in large farms. However, financial incentives must be adequate to enable farmers to viably produce veal (Becker & van Aken, 2021).

A second explanation for the high AMU on the veal farms might be the minimal risk tolerance of veal farmers concerning potentially high losses due to BRD which are not only caused by fatalities but also the markedly reduced DWG in calves after a severe episode of BRD. Experienced farmers rely heavily on repeated use of antimicrobials although in many cases their administration can be avoided either by waiting or by using exclusively non-steroidal anti-inflammatory drugs (Mahaendran et al., 2017).

## Conclusions

Preconditioning calves on dairy farms by intensified feeding, parenteral application of selenium, Vitamin E and iron, and vaccination against BRD resulted in a 10 kg higher BW compared to the controls at an age of roughly 30 days when the calves were transported to the veal farm. While the DWG on the veal farm did not vary significantly, the duration of fattening was reduced by 10 days leading to considerable economic benefit for the veal calf farmer. Antimicrobial use was generally high but did not vary between treatment and control groups. Further studies are needed to characterize the potential of preconditioning of future veal calves on their farms of origin regarding long-term effects on health and performance during large-scale veal fattening.

## Ethical statement

The study was approved according to Swiss legal requirements by the cantonal veterinary office Aargau (Ag 75,706).

## CRediT authorship contribution statement

**Julia Rell:** Writing – review & editing, Writing – original draft, Project administration, Investigation, Funding acquisition, Formal analysis, Data curation. **Michael Walkenhorst:** Writing – review & editing, Writing – original draft, Supervision, Project administration, Methodology, Funding acquisition, Data curation, Conceptualization. **Mirjam Holinger:** Writing – review & editing, Writing – original draft, Software, Methodology, Formal analysis. **Corinne Bähler:** Supervision, Methodology, Data curation, Conceptualization. **Jens Becker:** Writing – original draft, Methodology, Formal analysis. **Martin Kaske:** Writing – review & editing, Writing – original draft, Supervision, Resources, Methodology, Funding acquisition, Conceptualization.

## Declaration of competing interest

The authors declare that they have no known competing financial interests or personal relationships that could have appeared to influence the work reported in this paper.

## Appendix Supplementary materials

Image, application 1

Image, application 2
