# Supplementary material for: Effects of an optimized dairy calf-rearing protocol on performance and health in the subsequent fattening period on Swiss veal farms
Source: Vet Anim Sci. 2026 Jan 30;31:100590. doi: 10.1016/j.vas.2026.100590 (PMC12983269; doi:10.1016/j.vas.2026.100590)
Supplement: Supplementary file 1 [file mmc1.docx]

The treatment of individual animals is carried out according to the therapy plan

|  | **Initial treatment*** | | **Follow-up treatments** (medication and dose remain the same) | | | | | | | | | With-drawal period |
| --- | --- | --- | --- | --- | --- | --- | --- | --- | --- | --- | --- | --- |
|  | Medication, Dose | Day I | Day 2 | Day 3 | Day 4 | Day 5 | Day 6 | Day 7 | Day 8 | Day 9 | Day10 |  |
| Vitality/Fever/Respiratory tract | **Nuflor** (1ml/15kg s.c.) three times at intervals of 48 hours  **Metacam** (1 ml/40kg s.c.) on one to three consecutive days | Nu1 |  | Nu2 |  | Nu3 |  |  |  |  |  | after i.m.: 30 days  after s.c.: 44 Days |
|  |  | Meta1 | (Meta2) | (Meta3) |  |  |  |  |  |  |  | 15 Days |
| Gastrointestinal tract | **Rehycalb plus** ((1 sachet dissolved in 2 litres of warm water) by sucking bottle/bucket three times a day on day 1, and once a day on days 2 and 3  **If there is no clear improvement in the consistency of the faeces within 3 days:**  **R3 Rot Stricker** (½sachet) once a day in the mouth on the following four days  **Metacam** (1 ml/40kg s.c.) only once | Rehy1 | Rehy2 | Rehy3 |  |  |  |  |  |  |  | - |
|  |  |  |  |  | RS1 | RS2 | RS3 | RS4 |  |  |  | 4 Days |
|  |  |  |  |  | Meta1 |  |  |  |  |  |  | 15 Days |
| Ears | **Cyclosol** (10 ml/100 kg i.m. oder s.c.) three to four times at intervals of 72 hours  **Metacam** (2,5 ml/100 kg s.c.) on one to three consecutive days | Cyc1 |  |  | Cyc2 |  |  | Cyc3 |  |  | (Cyc4) | 14 Days |
|  |  | Meta1 | (Meta2) | (Meta3) |  |  |  |  |  |  |  | 15 Days |
| Navel | **Duphamox** (10 ml/100kg s.c.) four times at intervals of 48 hours  With a hunched back:  **Metacam** (2,5 ml/100 kg s.c.) on one to three consecutive days | Du1 |  | Du2 |  | Du3 |  | Du4 |  |  |  | 21 Days |
|  |  | Meta1 | (Meta2) | (Meta3) |  |  |  |  |  |  |  | 15 Days |
| Musculoskeletal system | Individually by arrangement |  |  |  |  |  |  |  |  |  |  |  |

* the initial treatment is carried out and entered on the health protocol, and the follow-up treatments are entered on the protocol sheets for the following days so that they only need to be ticked off
