# Supplementary material for: Effects of an optimized dairy calf-rearing protocol on performance and health in the subsequent fattening period on Swiss veal farms
Source: Vet Anim Sci. 2026 Jan 30;31:100590. doi: 10.1016/j.vas.2026.100590 (PMC12983269; doi:10.1016/j.vas.2026.100590)
Supplement: Supplementary file 2 [file mmc2.docx]

**Key Findings**

| *Range of findings:* | **Entry in the score sheet (below):** | | |
| --- | --- | --- | --- |
|  | **no entry** | **+** | **++** |
| *Reduced vitality* | lively, cannot be restrained | calmer than normal, standing, can be easily restrained | lying down, cannot be found or can only be found with difficulty |
| *Deviations respiratory tract* | Respiratory rate not or only difficult to determine; nostrils obB, occasional cough at most | Respiratory rate less than or equal to 40/min, cough, tearing under the eye, possibly watery NA | Respiratory rate greater than 40/min, "pumping", purulent NA, frequent coughing |
| *Deviations gastrointestinal tract* | Clean anal region | Soiled anal region; soupy faeces | Watery or soupy faeces in the stream, clearly distended on the left or right, conspicuous pressing on faeces |
| *Deviations ears* | No abnormalities, active ear play | conspicuous scratching with hind limb mass on the ear, rubbing with head against wall; conspicuous head shaking | Painful face, ear drooping on one or both sides, rotating movement (running in circles), "chattering" when the auricle is palpated |
| *Deviations navel* | dry, not sensitive to pressure | circumferencial more than finger strong, not painful | moist, painful, circumferencial more than finger strong |
| *Deviations musculoskeletal system* | inconspicuous | Stiff gait, no detectable lameness | Lameness on one or more limbs; conspicuous circumferencial on the joint |
| *Deviations temperature* | until 39,4°C | 39,5°C – 40,0°C | from 40,1°C on |

**Measure fever and note on the health record as soon as a finding (+ or ++) is entered**

**Treat if there is a + in two or more columns or ++ in at least one column**
